# Survival analysis of Rural Clinical School of Western Australia graduates: the long-term work of building a long-term rural medical workforce

**DOI:** 10.1186/s12913-019-4816-4

**Published:** 2019-12-26

**Authors:** Surabhi Gupta, Hanh Ngo, Tessa Burkitt, Ian Puddey, Denese Playford

**Affiliations:** Rural Clinical School of WA, School of Medicine, UWA, 35 Stirling Highway, Crawley, WA 6009 Australia

**Keywords:** Rural, Survival, Rural clinical school, Medical, Doctor, Workforce

## Abstract

**Background:**

Deficits in the rural medical workforce is an international issue. In Australia, The Rural Clinical School intervention is effective for initial recruitment of rural doctors. However, the extent of survival is not yet established. This paper summarises rural survival over a 10-year period.

**Methods:**

Rural Clinical School graduates of Western Australia were surveyed annually, 2006–2015, and post Graduate Years (PGY) 3–12 included. Survival was described as “tours of service”, where a tour was either a period of ≥1 year, or a period of ≥2 weeks, working rurally. A tour ended with a rural work gap of ≥52 weeks. Considering each exit from urban as an event, semi-parametric repeated measures survival models were fitted.

**Results:**

Of 468 graduates, using the ≥2 weeks definition, 239 PGY3–12 graduates spent at least one tour rurally (average 61.1, CI 52.5–69.7 weeks), and a total length of 14,607 weeks. Based on the tour definition of ≥1 year, 120 graduates completed at least one tour (average 1.89, 1.69–2.10 years), and a total of 227 years’ rural work. For both definitions, the number of tours increased from one to four by PGY10/11, giving 17,786 total weeks (342 years) across all PGYs for the ≥2 weeks tour definition, and 256 years total for ≥1 year. Significantly more graduates exited from urban work for the 2007–09 middle cohort compared with 2010–11 (HR 1.876, *p* = 0.022), but no significant difference between 2002 and 06 and 2010–11. Rural origin, age and gender were not statistically significant.

**Conclusions:**

PGY3–12 RCS graduates contributed substantially to the rural workforce: 51% did so by short rotations, while 26% contributed whole years of service. There was an apparent peak in entry and survival for the middle cohort and decline thereafter, likely attributable to lack of advanced/specialist vocational training. These data indicate a real commitment to rural practice by RCS graduates, and the need for rural vocational training as a key element of a successful rural survival strategy.

## Background

Lack of rural medical workforce is an issue in both developed [1, 2] and developing [3–5] nations. In Australia it is a particular issue, because the country is so strongly urbanised, with 71% of the population residing in major cities and just 2.2% living in remote or very remote Australia [6]. Distribution of practitioners from urban to rural and remote locations is an international problem. In 2015, Australia had 442 medical practitioners per 100,000 population in major cities, compared to 263 per 100,000 in remote and very remote areas [7]. The majority of specialists are urbanised with only 5% purely rural and 6% who commute between rural and urban [8].

To improve the distribution of medical practitioners multiple strategies have been implemented in Australia. The most visionary are those that seek to change medical students’ likelihood of choosing rural work. Undergraduate strategies include medical schools located wholly in rural areas - viz. James Cook University in Townsville, University of Wollongong and University of Newcastle [9]. Additional programmes of bonding and rural scholarships such as the rural Australian medical undergraduate scholarship scheme (RAMUS) and the medical rural bonded scholarships, have been given to rural students to study medicine on the basis of their higher probability of returning rural [10]. The Bonded Medical students’ Placement program requires that 28.5% of medical students, upon completion of their medical degree, work in districts of workforce shortage [11]. The John Flynn Placement Program is a briefer scholarship which selects students to be placed in a rural area repeatedly over a period of years to gain connection to a town. Finally, Rural Clinical Schools (RCS) offer extended clerkships to medical students in various rural locations Australia wide to experience rural medicine and rural life with the aim of subsequent recruitment to rural work [12].

The Rural Clinical School programme, established in 2002, places medical students in their penultimate year of study in rural areas Australia wide for a period of one to 2 years. The locations range in their degree of remoteness and the size of health services. There are multiple cross-sectional and cohort studies that highlight the effectiveness of rural clinical schools in workforce recruitment. The University of New South Wales has shown a three-fold increase in time spent rurally by RCS graduates from urban backgrounds [13]. The University of Queensland has shown that at the 5–7 year follow-up of RCS graduates, 40% have returned to rural areas [14], and that 18.8% of previous purely urban-trained students practiced rurally compared with 41.7% of RCS graduates [15]. The RCS of Western Australian (RCSWA) has shown a four-fold increase in the likelihood of working rurally after attending RCS [16], also highlighting that rural background graduates of RCSWA were most strongly associated with subsequently working rurally (OR 7.5, 95% CI[3.5, 15.8]). Similar data are supported by Kondalsamy-Chennakesavan, Eley, Ranmuthugala, Chater, Toombs, Darshan and Nicholson (2015) with regards to University of Queensland graduates. All these cross sectional studies on outcomes of RCS demonstrate that this immersion programme is an effective workforce strategy.

However, there are few data on the survival of these rural-working graduates. Kwan, Kondalsamy-Chennakesavan, Ranmuthugala, Toombs, Nicholson (2017) described a cohort of “long term rural stay” graduates, who spent more than 50% of training time in any rural area since graduation for 2002 to 2011 cohorts of UQ graduates [17]. .Predictors of long term rural work included attending RCS for one or 2 years (RCS-1 (OR 2.85 95%CI [1.77–4.58]), RCS-2 (OR 5.38 95% CI [3.15–9.20]), rural background (OR 2.10 95%CI [1.37–3.20]), bonded scholarship (OR 2.11 95% CI [1.19–3.76]) and becoming a General Practitioner (OR 3.40 95% CI [2.13–5.43]) [17]. These data appear to be more encouraging than the data reported by Playford, Qi-ng and Burkitt (2016) [18] who state that only 7% of graduates spent 75–100% of their of post-graduating time working in a rural area, while the majority spent up to 30% of their postgraduate training in a rural location [18]. However the Kwan et al. (2017) paper only reports on the 29% of all domestic medical graduates who responded to their survey and who therefore may represent alumni who are biased towards rural work [19].

Since both Kwan et al. (2017) and Playford et al. (2016) follow whole cohorts collectively (i.e., they did not follow individual graduates over time), they only partially contribute to the quantification of survival. These studies also did not take time since graduation into account.

Bailey, Wharton and Holman (2016) attempted to construct a specific measure of retention, by using “tours of service” to follow newly qualified General Practitioners (GPs) over a period of 10 years [20]. Tours of service were defined as rural location work with a break from rural work lasting no longer than a year. Two cohorts of GPs were followed: those who first commenced rural practice from 2004 to 2008 versus those in 2009–2013. This study showed that 41% of the 2004–2008 and 28% of the 2009–2013 cohort and cohort were not retained by end of the first year of fellowship. At 5 years, the survival rate for the cohort commencing 2004–2008 was 31% and was 38% for the cohort commencing 2009–2013 [20]. This definition of survival provides a useful statistic for graduates entering and exiting rural work.

The main aim of this study was to determine the number and duration of tours of service for RCS graduates overall, and by post-graduate year, asking whether this undergraduate programme is sufficient for long term rural work. We also assessed entry and survival in the rural workforce over time, and investigated possible contributing factors. These data make a contribution to the international evidence base on how to develop a sustainable rural medical workforce.

## Methods

### Participants

To be placed in RCS, undergraduate MBBS students went through an application and standardised interview process. If successful, they were distributed in groups of three to twelve to sites around STATE classified as Australian Geographical Classification – Remoteness Areas (ASGC-RAs) 2–5 [21]. They remained in a longitudinal integrated clerkship for one academic year in their penultimate year of study.

Participants for this study included all RCS graduates from The University NAME and the University NAME, who completed their penultimate year of medical school from 2002 to 2011, and responded to an annual contact either by survey or by phone. The contact survey contained information regarding practice location, college affiliation and years since graduation. Consenting graduates who did not respond to five consecutive emails were followed-up with up to five phone contacts.

### Data definitions and study variables

The outcome variables were the number of “tours of (rural) service” and their duration. ‘Rural’, RA2–5, was defined as all areas outside the capital city, versus ‘Urban, RA1, the urban city. “Tours of Service” were defined similarly to those described by Bailey et al (2016). However, for the purpose of this study, since early career graduates frequently entered and exited rural periods of training, two definitions of a ‘tour’ of service were utilised: (a) a period of at least two weeks within one calendar year (the less stringent definition, and the smallest interval of data collected); and (b) at least one year spent working in a rural area (the more stringent definition) with an end of a tour defined as a period of at least 52 consecutive weeks spent out of a rural area. It is noted that tour (a) is constrained within one calendar year, and there is no constraint on ‘breaks’ of service within that calendar year; whereas tour (b) can span over several consecutive calendar years. As such, multiple short tours in (a) within one calendar year are aggregated together in duration as one single tour for that year. The less stringent (≥2 weeks) tour definition captured what could be termed “frequent fliers” to rural locations. The term used in the Australian industrial sector for this kind of pattern is fly in – fly out, which comprises a core workforce model that refers to repeated visits to the same towns to supply ongoing service [22].

The independent variables included age at commencement year of RCS (Age: <25 years versus ≥25 years), gender, rural background, and RCS cohort (earliest 2002–2006, middle 2007–2009, versus most recent 2010–2011). Rural Background was defined as graduates with their principal home address in an RA 2–5 location for a period of at least five cumulative years before the commencement of medical school.

All information from the RCS longitudinal tracking project was entered into an Excel spreadsheet, which commenced with the first RCS clerkships in 2002. All graduates were followed from their third to twelfth Post Graduate Year (PGY), from 2006 to 2015. Graduates were thus contacted multiple times.

Some graduates took time out after graduation, and so were out of synchrony with their cohort; however to be consistent with our definition, they were included with the rest of their cohort.

The number of rural tours and the duration of the tours were calculated for each definition, for each graduate. Multiple tours could occur for the same participant during the study period of 2006–2015.

### Statistical analysis

For the survival analysis, original data records with participants’ data, available in separate rows for separate yearly follow-ups, were arranged in a Counting Process format such that the data for each row reflected a ‘continuous’, uninterrupted, event (instead of a year) [23, 24]. An example of these data arrangements is provided in Table 4 in Appendix.

Survival analysis of data for the one-year tour definition was conducted using SAS PROC PHREG, for semi-parametric repeated-measure data [25]. The robust/sandwich variance estimator output from the proportional means model was used. Survival analysis was performed for the follow–up period of 2011 to 2015, taking into account four potential contributing factors, namely, age, gender, rural background, and RCS cohort, as stated earlier. Kaplan-Meier survival curves were plotted for statistically significant effects, with ‘survival’ representing urban practice, and entry into rural work was considered ‘an event’. The baseline was assumed to be urban, because at the time of the data collection all new graduates in Western Australia had to start their medical career with an urban internship.

Missing points of data regarding rural location were censored as a non-event (i.e., equivalent to “Urban practice”, consistent with the Counting Process data format for repeated-measure survival data [16, 17]. This also gave the most strongly conservative measure of tours of service.

### Ethics approval

This study was approved by the University of NAME Human Research Ethics Committee RA/4/1/1627.

## Results

### Description of study sample

Twenty graduates did not consent to the longitudinal survey and yearly follow-ups, hence were not included in the analysis. Of the total of 468 consenting graduates included in the analysis; 278 graduates had no missing data. Of the remaining 190 graduates, 56 had one missing data point and 82 had two missing data points with 52 having three or more missing data points. There were 88 graduates from post-graduate years three to eight with all missing data points throughout the study who were conservatively coded as in urban practice, as those who did not respond to the surveys were more likely to be in urban work [19].

At the commencement of their rural clinical school the majority of participants were female (64%) and aged less than 25 years (71%) (Table 1). One-fifth (99) of graduates had a rural background. Approximately one-quarter (120–26%) were from the earliest (2002–06) cohort, one-third (196–32%) from the middle cohort (2007–09) and the remainder (42%) from the most recent cohort (2010–11).

**Table 1:** Key characteristics of study sample, stratified by RCS cohort

Demographics	RCS cohort	Categories	n	%	95% Confidence Interval
Age	2002–2006 (*n* = 120)	<25 years	102	85.0%	(78.6, 91.4%)
	2007–2009 (*n* = 196)	<25 years	138	70.4%	(64.0, 76.8%)
	2010–2011 (*n* = 152)	<25 years	93	61.2%	(53.4, 68.9%)
Gender	2002–2006 (*n* = 120)	Female	68	56.7%	(47.8, 65.5%)
	2007–2009 (*n* = 196)	Female^a^	130	66.3%	(59.7, 72.9%)
	2010–2011 (*n* = 152)	Female^a^	100	65.8%	(58.2, 73.3%)
Rural background	2002–2006 (*n* = 120)	Yes^a^	28	23.3%	(15.8, 30.9%)
	2007–2009 (*n* = 196)	Yes^b^	46	23.5%	(17.5, 29.4%)
	2010–2011 (*n* = 152)	Yes	25	16.4%	(10.6, 22.3%)

^a^Missing data for 1 graduate in each cohort

^b^Missing data for 2 graduates in this cohort

#### Rural work: Tours of service with two-week inclusion criterion

Counting all rural work of at least 2 weeks duration, a total of 17,786 weeks were spent rurally by 239 graduates from 2006 to 2015, equating to 342 years completed by 51% of graduates. For this less rigorous definition of rural work, the mean tour duration of the first tour was 61.1 (52.5, 69.7) weeks. Of these, 49 worked rurally more than once; 198 graduates had only 1 tour. These data are shown in Table 2.

**Table 2:** Total and mean duration (in weeks) in rural practice by Tour of service and Postgraduate year (PGY)^b^

Tour No.	Tour’s Duration (wks)	PGY										
	Mean (95% CI)^c^	3	4	5	6	7	8	9	10	11	12	Total
		(*n* = 468)	(*n* = 393)	(*n* = 316)	(*n* = 243)	(*n* = 178)	(*n* = 120)	(*n* = 85)	(*n* = 54)	(*n* = 28)	(*n* = 7)	(*N* = 468)
1 (*n* = 239) ^a^	61.1 (52.5, 69.7)	3375	3587	2868	1970	1462	712	295	182	104	52	14,607
2 (*n* = 41) ^a^	62.7 (45.6, 79.7)	0	0	452	710	465	366	260	118	130	68	2569
3 (*n* = 7) ^a^	85.4 (18.6, 152.2)	0	0	0	0	104	200	104	104	86	0	598
4 (*n* = 1) ^a^	12	0	0	0	0	0	0	0	12	0	0	12
Across All 288 Tours	61.8 (54.1, 69.4)	3375	3587	3320	2680	2031	1278	659	416	320	120	17,786
Overall Mean Duration per graduate per PGY^d^		7.2 (5.8, 8.6)	9.1 (7.4, 10.9)	10.5 (8.4, 12.6)	11.0 (8.5, 13.6)	11.4 (8.4, 14.4)	10.7 (7.3, 14.0)	7.8 (4.0, 11.5)	7.7 (3.1, 12.3)	11.4 (3.9, 19.0)	17.1 (−1.0, 35.3)	38.0 (32.1, 43.9)

The cohort size (n) per PGY in italic subheading only contributes to the calculations of the overall mean duration contributed by each graduate in each PGY (i.e., the final row of the table)

^a^Of *N* = 468, there were 229 graduates who did not contribute any tour (of at least 2 weeks consecutively) of rural service. The remaining 239 graduates had at least 1 tour, with 41 at least 2 tours, 7 with at least 3 tours, and 1 with 4 tours. Worded differently, 198 graduates had only 1 tour, 34 with 2 tours, 6 with 3 tours, and 1 with 4 tours

^b^A ‘tour’ of rural service here is defined as a duration of at least 2 consecutive weeks. Multiple short tours (of ≥2 consecutive weeks each) within one calendar year are summed together for duration calculation and treated as 1 tour for that particular year

^c^Summary tour statistics are calculated among graduates incurring the concerned tours of service only. For example, Tour 1’s duration is calculated based on *n* = 239 graduates who contributed at least 1 tour of rural service (of at least 2 consecutive weeks), and excludes *n* = 229 graduates with zero tour in rural work

^d^Statistics presented are Mean Duration (95% Confidence Interval), in weeks

Those who were more recently graduated (PGY3–6) had fewer instances of tours than older graduates (PGY7–12). The mean length of all tours in this definition was approximately 62 weeks, or 1.2 years.

#### Rural work: Tours of service with 1-year inclusion criterion

Counted as years spent rurally, 120 graduates (25.6%) completed at least one rural tour, with a mean tour length of 1.89 (1.69–2.10) years. Of these, 16 graduates completed more than 1 tour as shown in Table 3. Based on this more stringent criterion, a total of 256 years were spent rural by RCS graduates in PGY 3–12 from 2006 to 2015.

**Table 3:** Total and mean duration (in years) in rural practice by Tour of service and Postgraduate year (PGY)^b^

Tour No.	Tour’s Duration (yrs)	PGY										
	Mean (95% CI) ^c^	3	4	5	6	7	8	9	10	11	12	Total
		(*n* = 468)	(*n* = 393)	(*n* = 316)	(*n* = 243)	(*n* = 178)	(*n* = 120)	(*n* = 85)	(*n* = 54)	(*n* = 28)	(*n* = 7)	(*N* = 468)
1 (*n* = 120) ^a^	1.89 (1.69, 2.10)	41	48	48	41	26	12	6	3	2	0	227
2 (*n* = 11) ^a^	1.73 (1.08, 2.38)	0	0	2	3	5	3	2	2	1	1	19
3 (*n* = 4) ^a^	2.25 (0.78, 3.72)	0	0	0	0	1	2	3	1	1	1	9
4 (*n* = 1) ^a^	1	0	0	0	0	0	0	0	0	1	0	1
Across All 136 Tours	1.9 (1.7, 2.1)	41	48	50	44	32	17	11	6	5	2	256
Overall Mean Duration per graduate per PGY^d^		0.09 (0.06, 0.11)	0.12 (0.09, 0.15)	0.16 (0.12, 0.20)	0.18 (0.13, 0.23)	0.18 (0.12, 0.24)	0.14 (0.08, 0.20)	0.13 (0.06, 0.20)	0.11 (0.03, 0.20)	0.18 (0.03, 0.32)	0.29 (−0.08, 0.65)	0.55 (0.44, 0.65)

The cohort size (n) per PGY in italic subheading contributes to the calculations of the overall mean duration contributed by each graduate in each PGY (i.e., the final row of the table)

^a^Of *N* = 468, there were 348 graduates who did not contribute any tour (of at least 1 year consecutively) of rural service. The remaining 120 graduates had at least 1 tour, 11 with at least 2 tours, 4 with at least 3 tours, and 1 with 4 tours. Worded differently, 109 graduates had only 1 tour, 7 with 2 tours, 3 with 3 tours, and 1 with 4 tours

^b^A ‘tour’ of rural service here is defined as of at least 1 full calendar year (52 weeks) continuous duration

^c^Summary tour statistics are calculated among graduates incurring the concerned tours of service only. For example, Tour 1’s duration is calculated based on *n* = 120 graduates who contributed at least 1 tour of rural service (of at least 1 full calendar year long), and excludes *n* = 348 graduates with zero tour in rural work

^d^Statistics presented are Mean Duration (95% Confidence Interval), in years

The mean number of tours per person increased from PGY3 to PGY 12. The mean duration of tours also increased.

#### Survival analysis

**Fig. 1:**
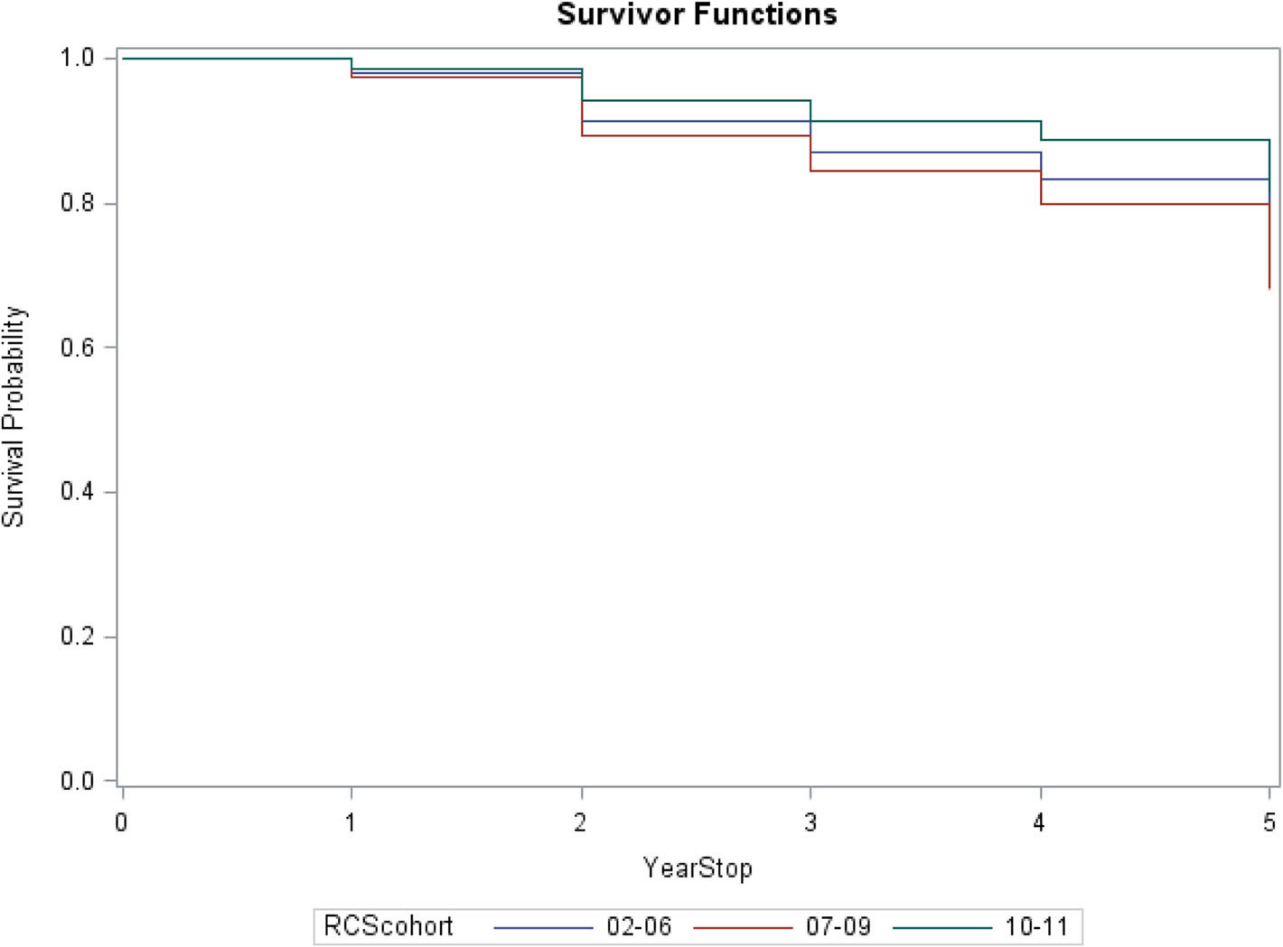
Probability of survival in urban work for three RCS cohorts, commencing ‘2002–2006’, ‘2007–2009’, and ‘2010–2011

Survival models were performed taking rural origin, age, gender and RCS year into account. The survival analysis curve showed a trend towards increasing exit from urban work (or increasing entry into rural work) (Fig. 1).

Background, comparing rural versus non-rural (hazard ratio of 1.118, *p* = 0.5555), age at commencement with RCS of <25 years versus ≥25 years (HR 0.749, *p* = 0.1039), and gender (HR 1.144 for males vs females, *p* = 0.4185) were not significant predictors of timing of entry into rural work.

RCS cohort year was a significant predictor of survival time, with a significantly higher rate of RCS graduates in the middle (2007–09) cohort leaving urban work (i.e. entering rural work), compared to the most recent cohort (2010–11), with a hazard ratio of 1.876 (*p* = 0.0220). The comparison for the earliest (2002–06) cohort versus the most recent did not reach statistical significance (HR 1.514, *p* = 0.1643). The Kaplan-Meier curves in Fig. 1 illustrate these observations.

## Discussion

We show that a substantial proportion of RCS graduates enter rural work, and collectively contribute to hundreds of years of rural service. These data stand in contrast with the work by Bailey et al. (2016) who describe a net loss from rural work over a period of 5 years for both newer and older GP fellows [20]. Russell et al. (2013) also show a decrease in rural work over time [26]. McGrail, Russell and Campbell (2016) used Generalised Estimating Equations (GEE) over successive cohorts of new rural GP fellows and also showed that within 5 years of follow-up, the proportion of GPs practicing rurally reduced each year [27]. The same time period of loss to rural work was found for newly graduating doctors from Thailand’s rural medical education project [28]. Similarly in America, even Rebinowitz’s robust data showed a declining survival curve for rural practice amongst both rural programme and no-rural programme graduates [29] One possible explanation for this discrepancy is that our RCS data describe graduates’ trajectory from the very beginning of their medical career, including stages before entering and/or fellowing in a vocational college, before urban specialty requirements are imposed. This selection of participants may comprise a period when new graduates are exploring work options, and before they have settled into later career patterns. If so, they are a powerful demonstration of the need for optimising rural career endpoints.

As this study fixed the baseline as urban work, since urban was where all graduates began their intern year, we have described loss to urban work, or gain to rural work, by an increasing number of graduates over time. By contrast, previous studies had a static cohort, with their baseline fixed in rural work, and so described loss to rural workforce over time. The present description of the net positive influence of an increasing pool of graduates on the rural workforce gives a direct estimate of RCS effect.

Using the one-year criterion, 25% of graduates did at least one tour of service of 1 year or more in their early postgraduate training. As a consequence, a total of 259 years were contributed to rural practice, which could be seen as a substantial commitment to rural work by early career RCS graduates. These data are in line with the finding from Russell et al. (2013) which showed the median stay of rural doctors - of unspecified vocational training level - was a period of 3 years [26], as was also the case in a study on rural survival of new graduates in Thailand [28]. The explanation for the relatively short stays in this study is that vocational training for these new graduates is highly likely to include mandatory urban rotations. Earlier work suggested that even post graduate year 2 work was unlikely for urban graduates in Australia [30].

As mentioned above, the less stringent (≥2 weeks) tour definition captured what could be termed “frequent fliers” to rural locations. The term used in the Australian industrial sector for this kind of pattern is fly in – fly out (FIFO), which comprises a core workforce model that refers to repeated visits to the same towns to supply ongoing service [22]. This kind of activity has not previously been captured for new medical graduates, and shows that a significant proportion (51.6%) of RCS alumni were spending multiple short term stays in rural practice from PGY3–12. Although the tour durations were limited, these data show considerable engagement in rural practice. The shorter stay criteria allowed identification of new work patterns which could not be obtained from the national registration board, the Australian Health Practitioner Regulation Agency, which only registers principal long term place of residence [31]. Our data suggest that a diverse set of definitions for rural practice, including FIFO models of practice, could be relevant to this newly developing rural workforce [32]. This kind of commitment to short visits sustained over time has been termed “RUFUS” in New Zealand, referring to “Rurally Focused Urban Specialist” [33].

These data confirm earlier work done by Playford et al. (2016), which showed considerable movement in and out of practice by RCS graduates [18]. There is some further evidence that mobility in the rural workforce is true for rural doctors in general [18]. McGrail et al. (2016) followed individual doctors in rural NSW and showed movement both between rural locations and back to the city [27]. It may be that mobility is attractive to these individuals, but it is more likely that there are not enough training opportunities rurally [27]. On a speculative level, these data suggest that given an increase in rural training opportunities, RCS graduates appear disposed to take them.

The survival analysis showed a significantly greater move out of urban practice into rural practice for RCS middle cohort (2007–2009) versus the latest (2010–2011) and earliest (2002–2006) cohorts. This is likely because the reality of post graduate training in STATE at the time of this study was that there were limited opportunities to work and train rurally. There were no year long rural internships, probably explaining the relatively lower work in the most recent cohort. There were also few rural vocational training options. This may explain why there were fewer working graduates in the older cohort. In contrast, graduates in the middle (2007–2009) cohort were at the stage of early college training in 2015 and so were able to complete some, but not all, terms of college training rurally, explaining why they had a relatively higher proportion of total training time in a rural setting.

Our data show that more than 50% of this RCS cohort contributed FIFO service, and that 26% contributed whole years of service. Although these statistics collectively may seem modest, we have shown previously that RCS graduates contributed on average approximately twice more the duration in rural practice than non-RCS counterparts [34]. The peak of service by PGY6, and decline thereafter, provides a powerful demonstration of the need for optimising rural career endpoints.

Earlier work by Rourke in Canada discusses the importance of rural tracks at all stages of training [35]. In Australia, Eley, Synnott, Baker and Chater (2012) report qualitative data for the University of Queensland RCS students which show that prolonged rural involvement during specialist training is associated with greater likelihood of long term rural work and rural life-decisions [14]. Recent opportunities and initiatives in some states – for example the extended training tracks in Queensland [36], clearly show the workforce impact of early and sustained recruitment into the rural workforce. To this end, the recent implementation of Integrated Rural Training Hubs in Australia has allowed a new focus on postgraduate rural training pathways, and so which is likely to prove significant to both early and sustained rural work after graduation.

In our sample, rural background was not associated with timing of rural work entry. This might mean that attracting any graduate into rural practice may have a positive effect. This observation agrees with the findings of McGrail et al. (2016) that any rural training is associated with sustained higher levels of rural work [27]. However, the fact that our data are agnostic with respect to the benefits of rural background, may also be due to the relatively small number of participants, which adds to the general consensus of RCS research, that we are at the earliest stages of being able to conduct large-scale longitudinal studies.

The survival analysis also showed that gender was a non-significant factor. This means that females and males were leaving urban for rural work at indistinguishable rates. This could be taken as a positive result because previous studies have shown that females are less likely to enter rural work [16].

There was also a lack of an age effect within this dataset which confirms prior RCS studies. Playford, Ngo, Gupta and Puddey (2017) showed that age was not an independent predictor for rural practice involvement [37].

### Limitations

This study included only RCS alumni, who presumably are already inclined towards rural work. It specifically intended to look at the work decisions of these graduates using two different criteria for rural work. Since other publications have looked at the control group of non-RCS relative to RCS graduates and shown very significant differences in work choices, we sought instead to look at the longevity of RCS graduates’ rural choices.

A significant minority of the data points had missing location of practice information, all of which were censored and conservatively coded as urban and included within the analysis. Therefore, some graduates, who were potentially rurally located but did not respond to the survey, were coded as urban. The means that the design of our study was biased against coding for rural work, and so that our positive results are likely to be a minimum estimate.

Our conservative survival analysis only included graduates who worked for at least one full year, which also likely biased our data against rural work.

## Conclusion

In conclusion, we found that RCS graduates undertake a significant amount of rural work from PGY 3–12, making it a workforce strategy worthy of consideration internationally. However, the relatively low rates of sustained rural practice in this historic sample suggests that post graduate initiatives are also required. To this end, recent funding to RCSs to increase postgraduate rural training opportunities in rural Australia may permit this new graduate workforce to further invest in long term rural career choices. The FIFO nature of some graduates’ rural work also suggests new modalities of what can be considered “rural work”. These data are useful in considering long term solutions to developing rural medical workforce.

## Data Availability

The datasets used and/or analysed during the current study are available from the corresponding author on reasonable request.

## Appendix

**Table 4:** Illustrations of data arrangements

Participant	Original format		New format for SAS PROC PHREG analysis		
	CollectionYear	RuralPractice	StartYear	EndYear	CensorStatus
1	2011	No	0	2	1
1	2012	No			
1	2013	Yes	2	4	2
1	2014	Yes			
1	2015	No	4	5	1

Calendar years were converted to simple time (yearly) units, with 0 indicating (end of) year 2010, and 5 indicating (end of) year 2015

This was necessary to preserve the correct duration, or the number of years, that the participatnt contributed to the study. For example, the maximum time period in this study is 5 years (2011 through 2015 inclusive). However, 2015–2011 (or in code 5–1) equals 4 years only

CensorStatus = 1 indicates censoring or a non-event (ie no rural practice). CensorStatus = 2 indicates an event (ie rural practice)

Grey-shaded rows of data did not exist in the new format

## Abbreviations

ASGC-RA: Australian Geographical Classification – Remoteness Areas
FIFO: Fly In – Fly Out
MBBS: Bachelor of Medicine, Bachelor of Surgery
PGY: Post Graduate Year
RCS: Rural Clinical School
RCSWA: Rural Clinical School of Western Australia
