# Publisher Correction: Survival analysis of Rural Clinical School of Western Australia graduates: the long-term work of building a long-term rural medical workforce

**DOI:** 10.1186/s12913-020-05210-9

**Published:** 2020-04-24

**Authors:** Surabhi Gupta, Hanh Ngo, Tessa Burkitt, Ian Puddey, Denese Playford

**Affiliations:** Rural Clinical School of WA, School of Medicine, UWA, 35 Stirling Highway, Crawley, WA 6009 Australia

**Correction to: BMC Health Serv Res (2019) 19:998**

**https://doi.org/10.1186/s12913-019-4816-4**

In the original publication of this article [1], an error occurred during the production of this article in Table 2. The revised Table 2 is shown below:

**Table 2:** Total and mean duration (in weeks) in rural practice by Tour of service and Postgraduate year (PGY)^b^

Tour No.	Tour’s Duration (wks)	PGY										
	Mean (95% CI)^**c**^	3	4	5	6	7	8	9	10	11	12	Total
		(*n* = 468)	(*n* = 393)	(*n* = 316)	(*n* = 243)	(*n* = 178)	(*n* = 120)	(*n* = 85)	(*n* = 54)	(*n* = 28)	(*n* = 7)	(*N* = 468)
**1 (*n*= 239)**^**a**^	61.1 (52.5, 69.7)	3375	7	2868	1970	1462	712	295	182	104	52	14607
**2 (*n*= 41)**^**a**^	62.7 (45.6, 79.7)	0	0	452	710	465	366	260	118	130	68	2569
**3 (*n*= 7)**^**a**^	85.4 (18.6, 152.2)	0	0	0	0	104	200	104	104	86	0	598
**4 (*n*= 1)**^**a**^	12	0	0	0	0	0	0	0	12	0	0	12
**Across All 288 Tours**	61.8 (54.1, 69.4)	3375	3587	3320	2680	2031	1278	659	416	320	120	17786
**Overall Mean Duration per graduate per PGY**^**d**^		7.2 (5.8, 8.6)	9.1 (7.4, 10.9)	10.5 (8.4, 12.6)	11.0 (8.5, 13.6)	11.4 (8.4, 14.4)	10.7 (7.3, 14.0)	7.8 (4.0, 11.5)	7.7 (3.1, 12.3)	11.4 (3.9, 19.0)	17.1 (-1.0, 35.3)	38.0 (32.1, 43.9)

The cohort size (n) per PGY in italic subheading only contributes to the calculations of the overall mean duration contributed by each graduate in each PGY (i.e., the final row of the table)

^a^Of *N* = 468, there were 229 graduates who did not contribute any tour (of at least 2 weeks consecutively) of rural service. The remaining 239 graduates had at least 1 tour, with 41 at least 2 tours, 7 with at least 3 tours, and 1 with 4 tours. Worded differently, 198 graduates had only 1 tour, 34 with 2 tours, 6 with 3 tours, and 1 with 4 tours

^b^ A ‘tour’ of rural service here is defined as a duration of at least 2 consecutive weeks. Multiple short tours (of ≥2 consecutive weeks each) within one calendar year are summed together for duration calculation and treated as 1 tour for that particular year

^c^ Summary tour statistics are calculated among graduates incurring the concerned tours of service only. For example, Tour 1’s duration is calculated based on *n* = 239 graduates who contributed at least 1 tour of rural service (of at least 2 consecutive weeks), and excludes *n* = 229 graduates with zero tour in rural work

^d^ Statistics presented are Mean Duration (95% Confidence Interval), in weeks

The original articles have been updated. The publisher apologizes for the inconvenience caused to our authors and readers.
